# The effect of low back pain and lower limb injury on lumbar multifidus muscle morphology and function in university soccer players

**DOI:** 10.1186/s12891-020-3119-6

**Published:** 2020-02-12

**Authors:** Neil Nandlall, Hassan Rivaz, Amanda Rizk, Stephane Frenette, Mathieu Boily, Maryse Fortin

**Affiliations:** 1 Department of Health, Kinesiology & Applied Physiology, Concordia University, Montreal, Quebec Canada; 2 Department of Electrical & Computer Engineering, Concordia University, Montreal, Quebec Canada; 3 PERFORM Centre, Concordia University, Montreal, Quebec Canada; 4 Department of Diagnostic Radiology, McGill University Health Center, Montreal, Quebec Canada; 5 Centre de recherche interdisciplinaire en réadaptation (CRIR), Constance Lethbridge Rehabilitation Centre, Montreal, Quebec Canada

**Keywords:** Lumbar Multifidus muscle, Ultrasound imaging, Dual-energy X-ray absorptiometry

## Abstract

**Background:**

The lumbar multifidus muscle (LMM) plays a critical role to stabilize the spine. While low back pain (LBP) is a common complaint in soccer players, few studies have examined LMM characteristics in this athletic population and their possible associations with LBP and lower limb injury. Therefore, the purpose of this study was to 1) investigate LMM characteristics in university soccer players and their potential association with LBP and lower limb injury; 2) examine the relationship between LMM characteristics and body composition measurements; and 3) examine seasonal changes in LMM characteristics.

**Methods:**

LMM ultrasound assessments were acquired in 27 soccer players (12 females, 15 males) from Concordia University during the preseason and assessments were repeated in 18 players at the end of the season. LMM cross-sectional area (CSA), echo-intensity and thickness at rest and during contraction (e.g. function) were assessed bilaterally in prone and standing positions, at the L5-S1 spinal level. A self-reported questionnaire was used to assess the history of LBP and lower limb injury. Dual-energy x-ray absorptiometry (DEXA) was used to acquire body composition measurements.

**Results:**

Side-to-side asymmetry of the LMM was significantly greater in males (*p* = 0.02). LMM thickness when contracted in the prone position (*p* = 0.04) and LMM CSA in standing (*p* = 0.02) were also significantly greater on the left side in male players. The LMM % thickness change during contraction in the prone position was significantly greater in players who reported having LBP in the previous 3-months (*p* < 0.001). LMM CSA (r = − 0.41, *p* = 0.01) and echo-intensity (*r* = 0.69, *p* < 0.001) were positively correlated to total % body fat. There was a small decrease in LMM thickness at rest in the prone position over the course of the season (*p* = 0.03).

**Conclusions:**

The greater LMM contraction in players with LBP may be a maladaptive strategy to splint and project the spine. LMM morphology measurements were correlated to body composition. The results provide new insights with regards to LMM morphology and activation in soccer players and their associations with injury and body composition measurements.

## Background

Soccer is one of the most popular sports in the world. Soccer athletes are exposed to high loads to the spinal region, pelvic region and lower limbs. As such, they require above average motor skills and stability of the lumbopelvic region in order to maintain a proper level of dynamic control. Low back pain (LBP) and lower limb injury are among the most common injuries in elite soccer players, with a yearly LBP prevalence of 64% and lower limb injury rate during competition varying between ~ 18 to 80% [1, 2]. Stability of the lumbar spine plays a critical role in preventing and reducing the risk of LBP-related injury, and the importance of paraspinal muscle recruitment and coordination was highlighted in several biomechanical studies [3, 4]. Smaller lumbar multifidus muscle (LMM) size and greater side-to-side asymmetry were indeed linked to LBP and lower limb injury in elite athletes [5–9].

A proper function of the LMM is critical to maintain the integrity of the kinetic chain and distribute forces to the lower limbs and upper limbs [10]. Although MRI and ultrasound imaging studies have reported morphological changes (e.g. atrophy, asymmetry) and altered function of the LMM in athletes with LBP, literature findings remain controversial and suggest that such changes may be related to specific sports or level of competition. Specifically, smaller LMM cross-sectional area (CSA) was reported in elite soccer players with LBP [9], but no such difference was found in adolescent soccer players [11]. While smaller LMM CSA was also reported to be a strong predictor of lower limb injury in professional Australian Football League (AFL) players [5], this has not been investigated in soccer players. Furthermore, the association between LMM muscle characteristics and LBP (or lower limb injury) has not been examined in female soccer players. Lastly, seasonal variations in LMM morphology and function in soccer players also warrants further investigation, as they may have important clinical implications for the susceptibility of injury.

While it is well established that muscle morphology is influenced by anthropometric factors, such as age, sex, physical activity levels, and body composition, [12–15] body mass index (BMI) remains the most frequently used variable to adjust for inter-subject variability in both anthropometric and body composition differences. BMI is, however, a poor indicator of body composition, especially in athletic populations, due to its inability to differentiate between lean and fat mass. Very few studies have used dual-energy X-ray Absorptiometry (DEXA) to investigate the association between muscle morphology and body composition. Additional studies are needed to clarify the relationship between accurate measures of body composition and LMM morphology.

Given that LMM plays a key role in lumbopelvic control, a better understanding of LMM characteristics and their association with body composition, both in male and female athletes, as well as their implications in different sports and susceptibility to injury may provide valuable insight for preseason-screening assessment and more effective and targeted rehabilitation. Therefore, the purpose of this this study was to: 1) investigate LMM characteristics in male and female collegiate soccer players, and their potential association with LBP and lower limb injury; 2) examine the relationship between LMM characteristics and body composition measurements; and 3) to examine seasonal changes in LMM characteristics in soccer players. We have hypothesized that smaller LMM CSA will be associated with LBP and lower limb injury in male and female soccer collegiate athletes. We have also hypothesized that lean muscle mass and % body fat will be associated positively associated with LMM CSA and LMM echo-intensity (EI – indicator of muscle quality using the ultrasound brightness scale), respectively.

## Methods

### Participants

Twenty-seven soccer players (12 females, 15 males) from the Concordia University varsity teams volunteered to participate in this study and were assessed during the preseason (end of August and the beginning of September 2016). From these, a total of 18 players (11 females, 7 males) were available and reassessed at the end of the competitive playing season (mid-November 2016). All available players were invited to participate to maximize the sample size, and thus no a priori sample size calculation was made. The exclusion criteria included previous history of severe trauma or spinal fracture, previous spinal surgery, observable spinal abnormalities, as all of these can affect paraspinal muscle morphology and/or function. Pregnancy was also an exclusion criterion as undergoing a DEXA scan was a requirement of this study. The study was approved by the Research Ethical Committee of the Institution and by the Central Ethics Committee of the Quebec Minister of Health and Social Services. All players that participated in this study provided informed consent.

### Procedures

A self-administrated questionnaire was used to collect information on players’ demographics and history of LBP during at the preseason. LBP was defined as pain localized between T12 and the gluteal fold with or without leg pain [16]; players were asked to answer “yes” or “no” to the presence of LBP during the past 3-months prior to the assessment. A visual Numerical Pain Scale (NRS) was used to assess the average LBP intensity (e.g. 10 point scale; 0 = no pain, 10 = worst pain possible). Players were also asked to indicate the LPB location (e.g. centered, right side, left side) and duration (in months) at both time points. Finally, players were questioned about their history of lower limb injury within the past 12-months and to provide the injured body part, if applicable. Similarly, at the end of the competitive season, players completed a related questionnaire asking about whether they experienced or suffered a lower limb injury during the season.

### Ultrasound

LMM assessments were performed using a LOGIQ e ultrasound machine (GE Healthcare, Milwaukee, WI) with a 5-MHz curvilinear probe. The imaging parameters were kept consistent for all acquisitions (frequency: 5 MHz, gain: 60, depth: 8.0 cm). The reliability of ultrasound imaging to assess LMM size and thickness has been previously established (intra- and inter-rater reliability ICCs = 0.94–0.99 [17]. LMM thickness change measurement is also highly correlated to EMG activity (r = 0.79, *p* < 0.001) [18].

#### LMM measurements

Players were placed in a prone position, on a therapy table, with a pillow under their abdomen to minimize lumbar lordosis [17]. They were instructed to relax the paraspinal musculature, and the spinous process of L5 was palpated and marked on the skin with a pen prior to imaging. For the assessment of LMM CSA, acoustic coupling gel was applied to the skin and the ultrasound probe was placed longitudinally along the midline of the lumbar spine to confirm the location of the L5 level [18]. Then, the probe was rotated and placed transversally over the L5 spinous process for imaging. Transverse images at L5 level were obtained bilaterally to assess LMM CSA, except for athletes with larger muscles, where the left and right sides were imaged separately. A total of 3 images were captured and saved for each side. The L5 level was selected as the level of assessment based on a previous study in elite AFL players reporting that decreased LMM CSA and increased side-to-side asymmetry, at this level, was a predictor of lower limb injury [5].

LMM function (e.g. contraction) was then evaluated by obtaining thickness measurements at rest and during contraction via a contralateral arm lift. For the thickness measurement, the LMM was imaged in the parasagittal view, which allows for the visualization of the L5/S1 zygapophyseal joints. Players were instructed to relax, while 3 images of LMM thickness were captured bilaterally, at rest. Players were then instructed to perform a contralateral arm lift holding a handheld weight [based on players’ body weight 1) < 68.2 kg = 0.68 kg weight, 2) 68.2–90.9 kg = 0.9 kg weight, 3) > 90.9 kg = 1.36 kg weight] while raising the loaded arm 5 cm off the therapy table (shoulder was placed in 120° of abduction and elbow 90° of flexion), in order to induce a submaximal (~ 30%) LMM isometric contraction [17–19]. While performing this task, players were instructed to maintain the position for 3 s and hold their breath at the end of normal exhalation, in order to minimize the effect of respiration on the thickness measures. Each player first had a practice trial, followed by 3 repeated contralateral arm lifts on each side.

Similarly, LMM measurements were then obtained in the standing position. Players were asked to stand barefoot on the floor with their arms relaxed on each side [20]. To achieve a habitual standing posture, they were instructed to first march on a spot for few seconds and remain in the position where their feet landed [20]. LMM CSA and thickness measurements at rest were obtained using the same procedure as describe above. To contract the LMM in this position, players performed a contralateral arm lift with the shoulder placed in 90° of flexion, with complete elbow extension and wrist in a neutral position (palm facing down) [20]. The same handled weight as previously determined for the prone measurements was also used to perform this task. Players maintained the position for 3 s and first had a practice trial, followed by 3 repeated contralateral arm lifts on each side.

#### Images assessment

**Fig. 1:**
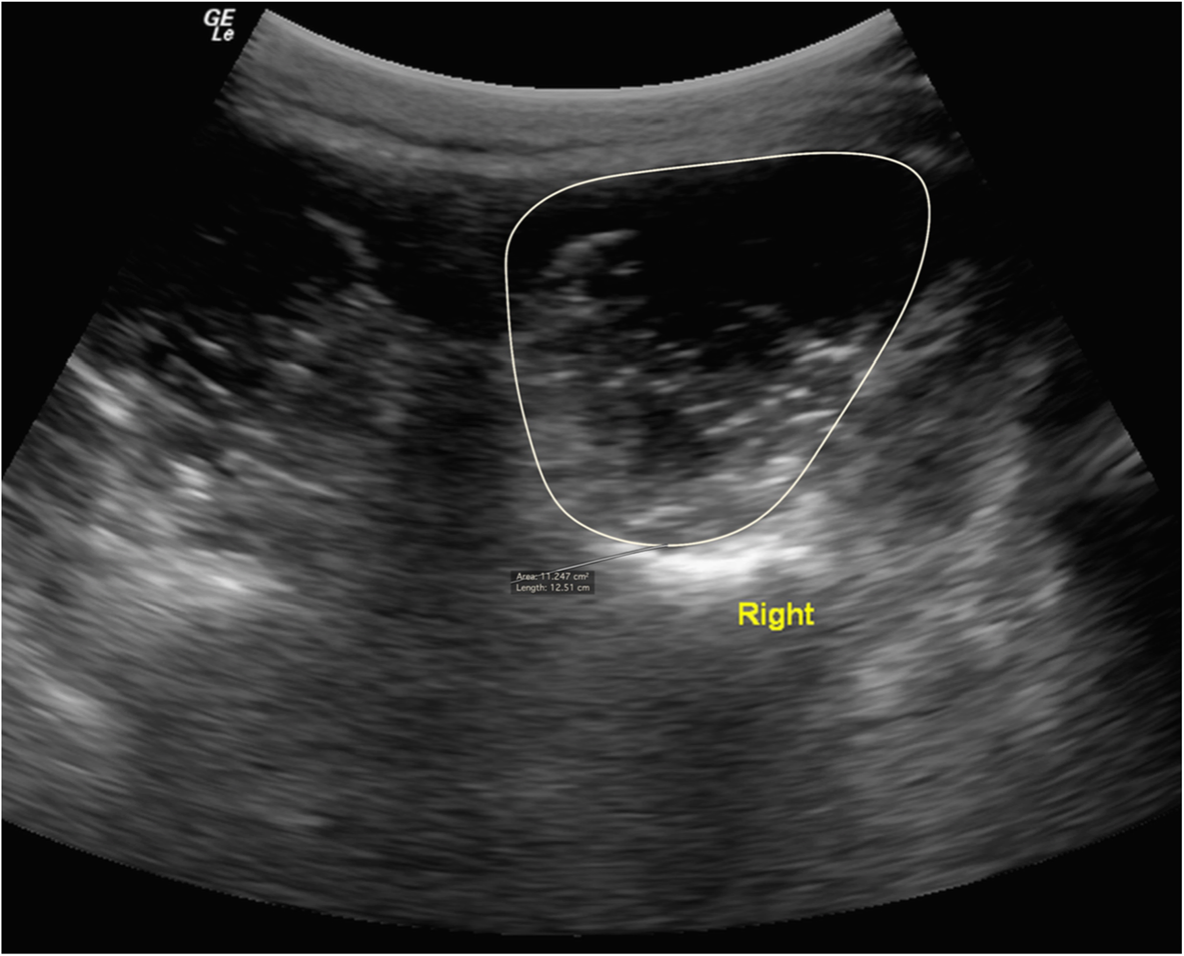
Lumbar multifidus muscle cross-sectional area (CSA) measurement in a male soccer player at the L5 vertebral level (prone position). The CSA measurement was also used to obtain echo-intensity measure in the prone position using the ImageJ histogram function

**Fig. 2:**
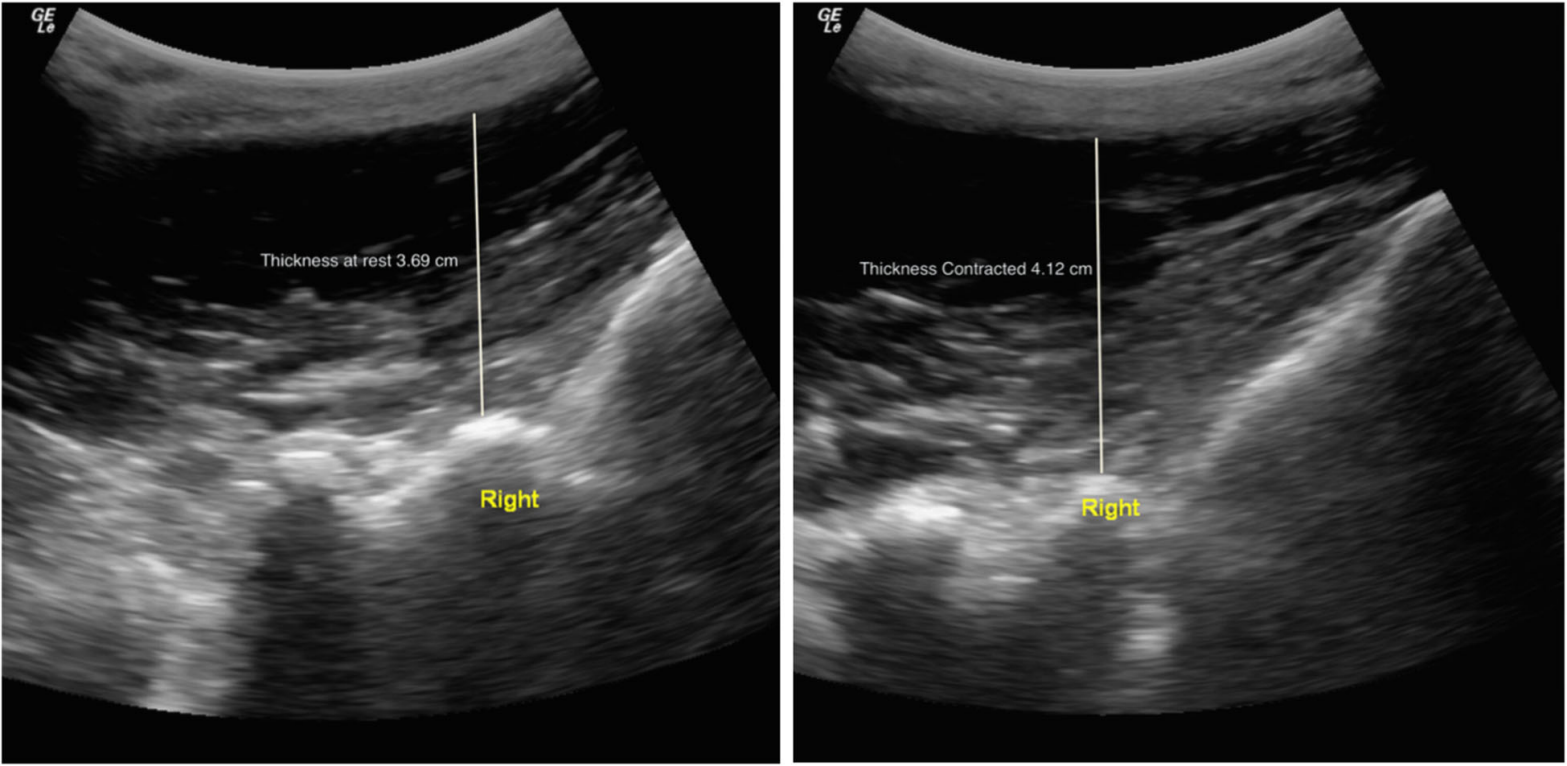
Lumbar multifidus muscle thickness measurement in at L5-S1, at rest (left image) and during contraction (right image) via a contralateral arm lift in a prone position

Ultrasound images were stored and analyzed offline using the OsiriX imaging software (OsiriXLiteVersion 9.0, Geneva, Switzerland). LMM CSA measurements were obtained by manually tracing the muscle borders on both sides, as showed in Fig. 1. The relative % asymmetry in LMM CSA between sides was assessed and calculated as follows: % relative asymmetry = [(larger side – smaller side)/larger side × 100]. The LMM thickness measurements (at rest and contracted) were obtained using linear measurements from the tip of the L5/S1 zygapophyseal joint to the inside edge of the superior muscle border (Fig. 2), in both the prone and standing positions. Each LMM measurement was obtained 3 times for each side, on 3 different images, and the average value was used for analysis. The following formula was used to assess the LMM contraction: thickness % change = [(thickness contraction – thickness rest)/thickness rest) × 100]. LMM EI was assessed using grayscale and standard histogram function (e.g. pixels expressed as a value between 0 (black) and 255 (white)) from the ImageJ software (National Institute of Health, USA, Version 1.49) [21]. Previous evidence confirmed that enhanced EI is indicative of a greater amount of intramuscular fat and connective tissue [22]. This measure was acquired by manually training the LMM region of interest (ROI), representing the CSA using the transverse ultrasound images obtained in the prone position, while avoiding the inclusion of surrounding bone or fascia. All LMM measurements were acquired by an experienced blinded researcher, with over 9 years of experience in spine imaging analysis. The rater also received prior training by a senior musculoskeletal ultrasound radiologist prior to the beginning of this study. The intra-rater reliability of the same rater for all LMM measurements (ICC_3,1_) was tested in a previous related study [23] and ranged between 0.96–0.99, 0.96–0.98 and 0.99 for the prone, standing and EI LMM measurements, respectively.

### DEXA

A full body DEXA scan (Lunear Prodigy Advance, GE) was obtained for each player and performed by a certified medical imaging technologist. All players removed any metal and were required to wear loose-fitting clothing, to avoid interference with the scan. The following information was entered into the system computer software prior to imaging: Age, height, weight, and ethnicity. Players were instructed to lie down supine in the center of the scanner, with their arms slightly away from the body, thumbs pointing upwards, and legs slightly apart with their toes pointing upwards. Total lean mass, total bone mass, total fat mass, and total percent body fat were acquired and used in the analysis.

### Statistical analysis

Means and standard deviations were calculated for players’ characteristics and body composition measurements. Paired t-tests were used to assess the difference in LMM characteristics between the right and left sides within male and female players, and analysis of variance (ANOVA) was used to assess the difference in LMM characteristics between male and female players. The associations between LMM characteristics, LBP and lower limb injury were initially examined using univariate linear regression. Height, weight, sex and total % body fat were then tested as possible covariates in multivariate analyses. These covariates were retained in the multivariable models only if they remained statistically significant (*p* < 0.05) or had a confounding effect (led to a ± 15% change in the beta coefficients of significant variables included in the multivariable model). Diagnostic plots (e.g. qq-plots and pp-plots) were used to evaluate the normality assumption*.* Finally, Pearson correlation and linear regression models were used to assess the relationship between LMM measurements of interest and body composition measurements. All analyses were performed with STATA (version 12.0, StataCorp, LP, College Station, Texas).

## Results

The players’ characteristics are presented in Table 1. The mean ± SD age, height, and weight was 20.4 ± 1.7 years, 172.3 ± 11.2 cm and 68.8 ± 8.7 Kg, respectively. The average number of years playing soccer at a competitive level was 8.5 years, and 1.4 years at the university level. A total of 30% (*n* = 8) reported LBP during the preseason (past 3 months) and 48% (*n* = 13) reported having a lower-limb injury in the past 12-months.

**Table 1:** Participants’ characteristics

	All (*n* = 27)	Female (*n* = 12)	Male (*n* = 15)
Age (yr)	20.4 ± 1.7	20.5 ± 1.6	20.3 ± 1.9
Height (cm)	172.3 ± 11.2	163.4 ± 8.5	179.5 ± 7.4
Weight (Kg)	68.8 ± 8.7	64.6 ± 8.2	72.1 ± 7.7
Total lean mass (kg)	52.2 ± 9.5	53.61 ± 4.1	59.1 ± 6.5
Total bone mass (kg)	3.1 ± 0.6	2.6 ± 0.3	3.4 ± 0.4
Total Fat mass (kg)	13.8 ± 5.9	18.6 ± 5.7	10.0 ± 2.3
Total body fat %	21.1 ± 8.8	29.4 ± 6.3	14.5 ± 2.9
BMI	23.2 ± 2.8	24.3 ± 3.4	22.4 ± 1.8
Dominant leg (n)			
Right	22	11	11
Left	4	1	3
Either	1	0	1
Soccer competitive level (yr)	8.5 ± 3.1	8.8 ± 2.6	8.3 ± 3.5
Soccer university level (yr)	1.4 ± 1.3	1.6 ± 1.2	1.3 ± 1.4
LBP preseason (n)	8	4	4
LBP location pre-season (n)			
Centered	1	0	1
Bilateral	2	1	1
Unilateral	5	3	2
LBP intensity (0–10 scale) preseason	4.3 ± 1.8	3.6 ± 1.9	5.0 ± 1.6
Lower body injury past 12-month	13	9	4
Lower body injury past 12-month body part			
Ankle	5	4	1
Thigh	4	4	0
Hip	3	1	2
Foot	1	0	1
LBP playing a season (n)*	5	2	3
LBP playing season location			
Centered	1	1	0
Bilateral	1	0	1
Unilateral	3	1	2
LBP intensity (0–10 scale) season	4.8 ± 2.2	3.5 ± 2.1	5.7 ± 2.1
Lower-body injury season (n)*	6	5	1
Lower-body injury season body part			
Ankle	4	3	1
Knee	2	2	0

*Data presented only for the 18 players that were reassessed at the end of the season

### LMM characteristics

LMM prone and standing measurements of the right and left sides, in female and male players are presented in Table 2. LMM CSA, thickness at rest and during contraction, both positions (prone and standing) were significantly greater in male as compared to female players. Side-to-side CSA asymmetry in the prone position was also significantly greater in males (*p* = 0.02). LMM EI was significantly greater in female (*p* < 0.001). There was no significant difference in the LMM % thickness change during contraction between male and female in prone or standing positions. LMM thickness contracted in the prone position and LMM CSA in the standing position was also significantly greater on the left side in male players (*p* = 0.04 and *p* = 0.02, respectively).

**Table 2:** LMM characteristics in female and male soccer players

PRONE	Female (*n* = 12)		Male (*n* = 15)	
	Right	Left	Right	Left
CSA (cm^2^)	**7.83 ± 1.29**	**7.91 ± 1.24**	**9.84 ± 1.17**	**10.03 ± 1.35**
CSA asymmetry (%)	**2.61 ± 1.54**		**5.00 ± 3.03**	
EI	**71.23 ± 17.79**	**70.71 ± 16.79**	**44.87 ± 14.87**	**44.91 ± 16.41**
Thickness (cm)				
Rest	**2.73 ± 0.42**	**2.79 ± 0.40**	**3.35 ± 0.47**	**3.38 ± 0.57**
Contracted	**3.13 ± 0.43**	**3.19 ± 0.35**	3.75 ± 0.48*	**3.85 ± 0.47**
% change	15.14 ± 7.06	14.88 ± 6.55	12.48 ± 9.03	15.02 ± 10.39
STANDING				
CSA (cm^2^)	**9.46 ± 1.81**	**9.63 ± 1.68**	11.33 ± 1.50*	**11.68 ± 1.66**
CSA asymmetry (%)	3.24 ± 3.25		3.93 ± 2.17	
Thickness (cm)				
Rest	**3.19 ± 0.37**	**3.24 ± 0.36**	**3.69 ± 0.60**	**3.74 ± 0.52**
Contracted	**3.25 ± 0.42**	**3.25 ± 0.37**	**3.88 ± 0.61**	**3.87 ± 0.58**
% change	2.98 ± 3.91	1.65 ± 5.26	5.21 ± 4.85	3.51 ± 4.71

bold = Significant difference (*p* < 0.05) between female and male players. * = Significant difference (*p* < 0.05) between right and left sides of female or male players

### LBP and lower limb injury comparisons

The % thickness change during contraction in the prone position was significantly greater in players who reported having LBP in the previous 3-months (*p* < 0.001, Table 3). While greater LMM thickness contracted was associated with having had a lower limb injury during the past 12-months (*p* = 0.03).

**Table 3:** Associations between LMM characteristics, low back pain, and lower limb injury

	LBP previous 3-months			Lower limb injury past 12-months		
	Coefficient	*P*-value	95% CI	Coefficient	P-value	95% CI
PRONE						
CSA (cm^2^)	−0.57	0.42	[−1.98, 0.85]	− 0.79	0.21	[−2.06, 0.48]
CSA asy (%)	− 0.28	0.82	[−2.68, 2.13]	− 0.22	0.84	[− 2.42, 1.98]
Thickness (cm)						
Rest	− 0.25	0.30	[− 0.73, 0.23]	− 0.05	0.81	[− 0.51, 0.40]
Contracted ^a^	0.07	0.75	[−0.40, 0.54]	**0.34**	**0.03**	**[0.04, 0.64]**
% change ^b^	**12.05**	**< 0.001**	**[7.63, 16.46]**	1.66	0.60	[−4.85, 8.19]
STANDING						
CSA (cm^2^)	−0.92	0.30	[−2.71, 0.87]	− 0.18	0.84	[−2.01, 1.65]
CSA asy (%)	−1.05	0.41	[−3.66, 1.56]	−0.88	0.46	[−3.3, 1.55]
Thickness (cm)						
Rest	−0.01	0.97	[−0.47, 0.45]	0.19	0.21	[−0.12, 0.51]
Contracted	0.01	0.97	[−0.52, 0.54]	0.13	0.13	[−0.08, 0.63]
% change	0.33	0.84	[−3.05, 3.70]	2.07	0.21	[−1.27, 5.43]

^a^ = Adjusted for weight and gender

^b^ = Adjusted for weight

### Associations between LMM characteristics and body composition

LMM muscle CSA was significantly correlated with height (prone: r = 0.52, *p* = 0.005; standing: r = 0.52, *p* = 0.01), weight (prone: r = 0.54, *p* = 0.003; standing: r = 0.55, *p* = 0.006), total bone mass (prone: r = 0.56, p = 0.003; standing: r = 0.51, *p* = 0.01), total lean mass (r = 0.65, *p* < 0.001; r = 061, *p* = 0.001). Similar significant correlations were also observed for LMM thickness at rest and LMM thickness during contraction in both positions. BMI was not correlated with LMM CSA in prone or standing (prone: r = 0.02, *p* = 0.91; standing: r = 0.01, *p* = 0.97) or LMM EI (r = 0.27, *p* = 0.16). LMM EI was correlated to total % body fat (r = 0.69, p < 0.001). Total % body fat was also correlated to LMM CSA in prone (r = − 0.41, *p* = 0.03).

### LMM seasonal changes

Variations in LMM characteristics over the course of the season were assessed in 18 available players. There were no significant changes in LMM CSA, side-to-side asymmetry, thickness during contraction or the % thickness change during contraction in the prone and standing positions between the pre-season and end-season measurements (Table 4). However, significant decrease in the thickness at rest in the prone position occurred during the season (*p* = 0.03). The changes between preseason and end-season LMM measurements were not associated with LBP during the season, but a greater decrease (atrophy) in LMM thickness at rest (prone position) over the course of the season was associated with having had a lower limb injury during the season (*p* = 0.01).

**Table 4:** Changes in LMM characteristics throughout the season (*n* = 18)

	Pre-Season	End-Season	%Change or Change
PRONE			
CSA (cm^2^)	8.52 ± 1.52	8.65 ± 1.48	1.54 ± 5.04%
CSA asymmetry (%)	2.87 ± 1.74	3.36 ± 3.56	0.49 ± 2.94
Thickness (cm)			
Rest	2.89 ± 0.41	2.83 ± 0.40	−2.14 ± 6.33
Contracted	3.32 ± 0.42	3.26 ± 0.45	−2.23 ± 5.71
% change	15.24 ± 6.04	15.50 ± 6.37	−0.12 ± 5.56
STANDING			
CSA (cm^2^)	10.12 ± 1.88	9.91 ± 1.57	−1.99 ± 8.18
CSA asymmetry (%)	3.43 ± 3.07	2.76 ± 2.42	−0.68 ± 1.77
Thickness (cm)			
Rest	**3.34 ± 0.35**	**3.26 ± 0.36**	−2.36 ± 4.45
Contracted	3.44 ± 0.42	3.41 ± 0.43	−0.88 ± 2.71
% change	3.49 ± 3.82	4.61 ± 3.87	1.49 ± 3.33

bold = Significant difference (*p* < 0.05) between pre-season and end-season measurements

## Discussion

As expected, male had greater LMM CSA compared to female soccer players. Our findings also suggest that male and female soccer players appeared to have larger LMM CSA at the L5 level than healthy non-athlete subjects of similar age [24]. Such hypertrophy is likely an adaptation related to the high-intensity, repetitive movements and specific functional demands of the sport. The LMM thickness when contracted and CSA while standing were also significantly greater on the left side as compared to the right in male athletes. As kicking is an asymmetrical and ballistic task [25] that involves hip flexion, trunk rotation and stabilization on the non-dominant leg [26, 27], this may have contributed to the greater LMM size on the left side. While this finding was also reported in collegiate ballroom dancers [28], other studies in elite athletes reported symmetrical CSAs [29, 30], as well as larger LMM CSA on the dominant (right) side [31, 32], suggesting that specialized movements and sport specific training effects likely influence LMM morphology [28].

In accordance with Fortin et al., a significant increase in LMM CSA was observed when measurements were obtained in the standing position [23]. This finding was also reported in non-athletic populations [33]. The sharp increase in LMM CSA in this position characterizes the role and increase of force exerted by the LMM to provide control and dynamic stability to the lumbar segments while standing upright [33]. As the LMM is largely responsible for compression load and dynamic stability at the lower levels of the spine when upright, future ultrasound studies should investigate LMM morphology and neuromuscular control in such functional and sport-related positions, as the ability to modulate LMM may have important implications for sport performance and susceptibility to injury.

We found no significant difference in LMM CSA between soccer players with and without LBP. This finding is in accordance with a previous study from Noormohammadpour et al. reporting no difference in LMM CSA at the L4 level, between asymptomatic adolescent soccer players and players who reported LBP during their sport life, during the last year, during the last month or those with LBP that increase during sport activity [11]. Conversely, Hides et al. showed that elite soccer players with LBP had significantly smaller LMM CSA at the L4 and L5 level, as compared to players without LBP [9]. The different results may relate to the level of competition, as well as features of the training regimen. While university level hockey players [23] and professional ballet dancers [34] with LBP also showed deficits in resting LMM CSA compared to their asymptomatic counterparts, other studies in athletes reported no such association [28–30]. The discrepancy in findings suggests that some athletic populations may behave differently with regards to LMM size, training effects and LBP [28].

Soccer players with LBP, however, had a greater contraction of the LMM in the prone position as compared to players without LBP. Hides et al. also reported greater LMM contraction (prone position) at the L2 level in professional soccer players with LBP [9], as well as greater contraction of the transverse abdominis (TrA) muscle. Similar findings were also reported in professional cricketers and non-athletic populations with LBP [35, 36]. Such increases in LMM and TrA activation is thought to represent a maladaptive strategy, resulting from movement and motor control impairments. Individuals with motor control impairments display deficits in lumbopelvic stability, which is manifested as a loss of control in the neutral zone and spinal motion segment, resulting in pain and disability [37]. Increased trunk muscular activation was also reported in subgroups of patients with non-specific chronic LBP (e.g. active extension motor control impairment and flexion pattern motor control impairment) when performing functional tasks as compared to healthy subjects, further suggesting that increased muscle co-contraction may be a factor for individuals with pain [38]. Persistent muscle activation may restrict interverbal motion as a protective mechanism of the neuromuscular system and thus allow a strategy to splint or stiffen the spine in order to protect dysfunctional passive spinal structure in provocative movements [38, 39].

Our findings suggest that LMM thickness when contracted in the prone position was slightly greater in players who reported having a lower limb injury in the past 12-months. To the best of our knowledge, we are not aware of any studies that have investigated the relationship between lower limb injury and LMM morphology and function in soccer players. However, smaller LMM CSA was found to be a strong predictor for lower limb injury in AFL players [5]. While Hides et al. reported asymmetry in hip adductor and abductor muscle strength in elite soccer players with LBP (e.g. stronger adductor muscles), the relationship with lower limb injury was not investigated [9]. Mueller at al. reported that individuals with LBP usually adopt a trunk flexed posture and walk with more extended knees, which could potentially increase the risk of lower limb injury [40]. Indeed, AFL players with LBP in the preseason were found to have a 98% increase in the odds of suffering a lower limb injury [5]. Interestingly, no difference in leg length discrepancy, hamstring flexibility, active lumbar forward flexion was reported between adolescent soccer players with and without LBP, but the relationship with lower limb injury was not investigated [11].

LMM CSA and thickness were significantly correlated with players’ height, weight, total bone mass and total lean mass in prone and standing. While the total % body fat was strongly correlated to LMM EI and LMM CSA, BMI was not. These findings are in accordance with a previous study in collegiate hockey players [23] and provide additional evidence to support that body composition cannot be ignored when assessing LMM morphology, especially in athletes. Additional related studies should consider using DEXA to assess body composition in athletes and how such measurements may influence muscle morphology, function, injury and performance in athletes.

With the exception of a slight decrease in the contracted LMM thickness while standing which is likely not clinically significant, our results revealed no significant changes in LMM morphology or function over the course of one season in collegiate soccer players. Hides et al., however, reported an increase in LMM CSA at the L4 and L5 levels in elite soccer players across the preseason, with the largest increased observed in players that reported LBP at the start of the preseason [9]. Importantly, the soccer players included in the latter study, however, also completed a preseason injury prevention training program targeting the LMM, which likely explains the observed positive changes in LMM size.

Few studies investigated the seasonal changes of trunk muscle involved in lumbopelvic control in athletes. Hides and Stanton reported a significant decrease in LMM CSA and increase in the erector spinae CSA and internal oblique thickness over the course of a competitive season in professional AFL players [41]. Such patterns of imbalance between the local and global muscles during the playing season can be problematic, as it may generate large unfavorable forces to the spine [41]. As our findings also revealed that a greater decrease in LMM thickness at rest (prone position) was associated with having suffered a lower limb injury during the playing season, additional studies should investigate seasonal variations in trunk muscles involved in lumbopelvic stability among elite athletes, as muscle atrophy, imbalance and neuromuscular deficits may contribute to the susceptibility of injury.

A limitation of this study is the relatively small sample size. Although comparable to other studies in elite athletes, [6, 9, 11, 23, 28–32] this study may be underpowered. Second, only 18 players were available for the end-season assessment. While this was mostly due to academic commitments as the end of the season was also in the exams period, this may have introduced selection bias. Lastly, we had no control group. However, methodological strengths of the current study consist of the inclusion of both, male and female soccer athletes, as well as the acquisition of DEXA body compositions measurements and LMM measurements in a standing position.

## Conclusions

Difference in LMM characteristics between male and female soccer players were observed. Soccer players with LBP in the previous 3-months had a greater contraction of the LMM in a prone position. While we observed minimal seasonal changes in LMM morphology and function, a greater decrease in LMM thickness was associated with having suffered a lower limb injury during the playing season. LMM characteristics were also correlated to body composition measurements. Preseason screening assessment of the LMM characteristics may be useful in an injury prevention program.

## Data Availability

The datasets used and/or analyzed during the current study are available from the corresponding author on reasonable request.

## Abbreviations

AFL: Australian Football League
BMI: Body Mass Index
CSA: Cross-Sectional Area
DEXA: Dual-energy X-ray absorptiometry
EI: Echo Intensity
LBP: Low Back Pain
LMM: Lumbar Multifidus Muscle
TrA: Transverse Abdominis muscle
